# Prevention of Sudden Cardiac Death in Hypertrophic Cardiomyopathy: What has Changed in The Guidelines?

**DOI:** 10.5935/abc.20180099

**Published:** 2018-07

**Authors:** Liliana Reis, Rogerio Teixeira, Andreia Fernandes, Inês Almeida, Marta Madeira, Joana Silva, Ana Botelho, João Pais, José Nascimento, Lino Gonçalves

**Affiliations:** Centro Hospitalar e Universitário de Coimbra, Serviço de Cardiologia, Coimbra - Portugal

**Keywords:** Death, Sudden Cardiac / prevention & control, Cardiomyopathy, Hypertrophic / complications, Defibrillators, Implantable / trends, Syncope, Diagnostic Imaging

## Abstract

**Background:**

The new European Society of Cardiology guidelines for hypertrophic cardiomyopathy (HCM) define the estimation of sudden cardiac death (SCD) risk as an integral part of clinical management. An implantable cardioverter defibrillator (ICD) is recommended (class IIa) when the risk is ≥ 6%.

**Objectives:**

To compare the SCD risk stratification according to the 2011 and 2014 recommendations for ICD implantation in patients with HCM.

**Methods:**

Retrospective study including 105 patients diagnosed with HCM. The indication for ICD was assessed using the 2011 and 2014 guidelines. Statistical analysis was performed using SPSS software version 19.0.0.2®. The tests performed were bilateral, considering the significance level of 5% (p < 0.05).

**Results:**

Regarding primary prevention, according to the 2011 ACCF/AHA recommendations, 39.0% of the patients had indication for ICD implantation (level of evidence IIa). Using the 2014 guidelines, only 12.4% of the patients had an indication for ICD implantation. Comparing the two risk stratification models for patients with HCM, we detected a significant reduction in the number of indications for ICD implantation (p < 0.001). Of the 41 patients classified as IIa according to the 2011 recommendations, 68.3% received a different classification according to the 2014 guidelines.

**Conclusion:**

Significant differences were found when comparing the SCD risk stratification for ICD implantation in the two guidelines. The current SCD risk score seems to identify many low-risk patients who are not candidates for ICD implantation. The use of this new score results in a significant reduction in the number of ICD implanted.

## Introduction

Hypertrophic cardiomyopathy (HCM) is characterized by left ventricular hypertrophy (LVH) not explained only by ventricular overload conditions.^1^ It is the most common cardiovascular genetic pathology, with an estimated prevalence in the general population of 1:500 individuals.^2,3^ Hypertrophic cardiomyopathy is a complex disease, regarding genetic diversity (for which, more than 1400 mutations have been identified in 11 different genes), phenotypic expression, histological characteristics and manifested symptoms.^4,5^

Sudden cardiac death (SCD) is the most unpredictable and devastating consequence of HCM, occurring mainly in young or asymptomatic individuals or those with frustrated symptomatology.^4-6^ Recent data have pointed to a 0.7%/year incidence of SCD, the total incidence of cardiovascular death being 1.4%/year.^7^ The exclusive efficacy of implantable cardioverter defibrillator (ICD) in the prevention of SCD is well known.^1,8,9^ When approaching patients with HCM and their families, the correct assessment of the SCD risk and potential benefit of implanting that device for primary prevention is fundamental.^1-3^

According to the American College of Cardiology Foundation/American Heart Association (ACCF/AHA) recommendations for the diagnosis and treatment of HCM published in 2011, the presence of at least one risk factor for SCD [maximal left ventricular (LV) wall thickness ≥ 30 mm, unexplained syncope, nonsustained ventricular tachycardia (NSVT), family history of sudden death and abnormal blood pressure response during exercise] is a class IIa recommendation for the implantation of ICD in primary prevention.^10^

However, a recent study by O'Mahony et al. has suggested that the use of those criteria overestimates the risk for SCD, resulting in the excessive and unnecessary implantation of ICD in a substantial percentage of patients, exposing them to unnecessary iatrogenic complications.^11^ In addition, those authors have concluded that the limited power in risk stratification results from the fact that the algorithm is based on a dichotomous classification of the risk variables.^11^ Thus, the risk factors are recognized to be non-static and to have a cumulative evolutionary potential, with corresponding increase in the likelihood of SCD.^12^

In 2013, a new mathematical model was proposed to estimate the individual risk of SCD at 5 years.^13,14^ That model, based on a retrospective study of a population of 3675 patients from six centers, comprises some classical risk factors combined with LV outflow tract gradient, left atrial diameter, and age, which are considered continuous variables.^13^ The following formula is used:

**Table:** 

**Probability of SCD at 5 years = 1 – 0.998 exp(prognostic index)** Prognostic index = [0.15939858 x maximal wall thickness (mm)] - 0.00294271 x maximal wall thickness^2^ (mm^2^)] + [0.0259082 x left atrial diameter (mm)] + [0.00446131 x maximal LV outflow tract gradient (rest/Valsalva - mm Hg)] + [0.4583082 x family history of SCD] + [0.82639195 x NSVT] + [0.71650361 x unexplained syncope] - [0.01799934 x age on clinical assessment (years)].

According to the literature, that score is more accurate to differentiate patients at low risk from those at high risk,^13^ and was incorporated into the most recent European Society of Cardiology (ESC) recommendations published in 2014 as a valid and independent method for risk stratification.^1^

The direct comparison of the discriminative value of the two risk score systems to identify patients requiring an ICD in a non-selected population with HCM has not been performed in Portugal.

This study aimed at comparing the risk stratification of SCD in a population of patients with HCM, according to the 2011 and 2014 recommendations, and at characterizing the clinical performance of the risk model of SCD due to HCM individually in a Portuguese population with HCM.

## Methods

### Population

Retrospective single-center analysis of patients diagnosed with HCM and regularly followed up at a cardiology outpatient clinic of one single tertiary center for 6 years. The definition of HCM was based on a wall thickness ≥ 15 mm in one or more LV myocardial segments, which was not explained only by LV overload, and measured by use of any imaging technique [echocardiography, cardiac magnetic resonance imaging (CMRI) or computerized tomography (CT)]. The clinical diagnosis of HCM in a first-degree relative of a patient with unequivocal disease (LVH ≥ 15 mm) is based on the presence of unexplained LV wall thickening ≥ 13 mm in one or more myocardial segments, measured by use of cardiac imaging techniques.^1-3,15,16^

This study included 109 patients with LVH. Those whose complementary study revealed hereditary metabolic and neuromuscular causes (2 patients with cardiac amyloidosis, 1 patient with Noonan syndrome and 1 patient with Anderson-Fabry disease) were excluded. The total sample of this study comprised 105 index patients diagnosed with HCM.

The indication for an ICD implantation was based on the 2011 ACCF/AHA recommendations, and the patients received an ICD when they had at least one risk factor for SCD, according to the 2011 guidelines.

Later, a new analysis was performed based on the current recommendations (2014 ESC), using the data of the patients at the time of the diagnosis. The current model of risk for SCD due to HCM is part of a predefined set of 7 potentially prognostic variables.^1^ By using an online calculator, a predictive risk score of SCD due to HCM at 5 years was generated. According to that value, patients were stratified into three risk categories for ICD implantation: < 4%/5 years (ICD usually not considered); 4% to 6%/5 years (ICD can be considered); > 6%/5 years (ICD should be considered).^1^

### Characteristics of the population base and complementary study

The following baseline characteristics were collected at the time of diagnosis: age, sex, arterial hypertension, diabetes *mellitus*, atrial fibrillation, unexplained syncope, history of SCD in a first-degree relative (< 40 years), New York Heart Association (NYHA) functional class.

All patients underwent initial 12-lead electrocardiography, with assessment of LVH voltage criteria, Q waves, left axis deviation and atrioventricular conduction disorders.

All patients underwent transthoracic echocardiography. The following parameters were recorded: LV diastolic diameter, LV wall thickness from base to apex, presence of LV outflow tract gradient at rest and after the Valsalva maneuver, left atrial diameter, classification of LV systolic (LV ejection fraction) and diastolic function. The LV outflow tract obstruction caused by the systolic anterior motion (SAM) of the mitral valve leaflets was defined as a peak pressure gradient at the LV outflow tract ≥ 30 mm Hg at rest or during physiological challenge.^1^ Twenty-five patients (23.8%) with no gradient at rest underwent exercise echocardiography to assess the presence of gradient during exercise.

All patients underwent 24-hour Holter at the initial assessment or during clinical follow-up, allowing the identification of ventricular extrasystoles and/or NSVT episodes, defined as the presence of at least three consecutive ventricular complexes, lasting less than 30 seconds and without hemodynamic impairment.

All patients underwent exercise test according to the Bruce protocol to assess blood pressure response during exercise. Anomalous response was defined as the lack of blood pressure increase by 20 mmHg or a decrease of at least 20 mmHg during exertion.

Cardiac magnetic resonance imaging was performed in 85 (80.2%) patients who had access to a magnetic resonance scanner 1.5 Tesla (Phillips®). The following parameters were recorded for analysis: left atrial area, greater LV wall thickness, LV ejection fraction and presence of late enhancement after intravenous gadolinium administration.

Screening for sarcomere protein gene mutation (*MYL2* and *MYL3* = myosin light chain 2 and 3; *MYBPC3* = myosin-binding protein C; *MYH7* = myosin heavy chain 7; *TNNI3* = cardiac troponin I; *TNNT2* = cardiac troponin T; *TPM1* = tropomyosin alpha-1 chain) was conducted in 83 patients (79.0%), and screening for Anderson-Fabry disease, in 76 patients (72.4%). The screening for Anderson-Fabry disease in men was based on dried blood spot (DBS) testing to assess galactosidase A (GLA) activity. When GLA activity was reduced (< 5%), a 10-mL blood sample was collected in an EDTA tube for further GLA gene sequencing at a medical genetic center. In women, GLA gene sequencing analysis was performed in an external laboratory to identify mutations.^17^ One patient was diagnosed with that disease, being excluded from the study.

### Statistical analysis

The numeric variables were expressed as means and standard deviations, and the categorical variables, as absolute and relative frequencies. Regarding the recommendations for ICD implantation in primary prevention, the comparison between the two guidelines was performed by use of the McNemar test. On the first analysis, we assumed that the 2014 ESC classification IIb does not usually recommend ICD implantation, therefore, that classification was grouped together with the recommendation level III. The potency of that test is 99.9%, considering: the significance level of 5%; sample size of 105; the 0.001 proportion of patients classified as III according to the 2011 guideline and as IIa according to the 2014 guideline; and the 0.28 proportion of patients classified as IIa according to the 2011 guideline and as IIb/III according to the 2014 guideline.

Later, four groups of patients were defined as follows: patients classified as III according to both 2011 and 2014 guidelines; patients classified as IIa according to the 2011 guideline and as III according to the 2014 guideline; patients classified as IIa according to the 2011 guideline and as IIb according to the 2014 guideline; and patients classified as IIa according to both 2011 and 2014 guidelines. Because one of the assumptions to apply the chi-square test with asymptotic distribution was not met, those groups were compared regarding the percentage of ICD implantation by use of the exact chi-square test.

It is worth noting that, given the size of the sample, its power was calculated, ensuring that the number of patients was sufficient to draw conclusions.

The statistical analysis was performed by using the SPSS software, version 19.0.0.2®. The tests performed were bilateral, and the significance level of 5% (p < 0.05) was adopted.

## Results

The study sample comprised 105 patients, 53% of whom were of the female sex, the mean age at the time of diagnosis being 58 ± 18 years. Table 1 shows the major characteristics of the population. The functional capacity on the initial assessment was as follows: 45 (42.8%) patients were asymptomatic (NYHA class I), 40 (38.1%) had mild symptoms (NYHA class II), and 9 (8.6%) had severe symptoms (NYHA classes III and IV).

**Table 1:** Major characteristics of the population

Personal antecedents	
Arterial hypertension	74 (70.5%)
Atrial fibrillation	34 (32.4%)
Family history of sudden cardiac death	18 (17.1%)
Type 2 diabetes *mellitus*	16 (15.2%)
Previous syncope	14 (13.3%)
Previous coronary artery disease	10 (9.4%)
**12-lead electrocardiogram**	
Criteria of LVH	69 (65.7%)
Left anterior hemiblock	25 (23.8%)
First-degree AVB	16 (15.2%)
Complete right bundle-branch block	7 (6.7%)
Complete left bundle-branch block	5 (4.8%)
**Transthoracic echocardiogram**	
Septal HCM	72 (68.5%)
Concentric HCM	17 (16.1%)
Apical HCM	15 (14.3%)
Obstructive HCM	43 (40.9%)
LVEF ≤ 40%	4 (3.8%)
Mitral regurgitation	
- Mild	55 (52.4%)
- Moderate	16 (15.2%)
- Severe	8 (7.6%)
**Exercise test**	
Hypotensive response to exertion	4 (3.8%)
**Cardiac magnetic resonance**	
LA area, cm^2^	43.6 ± 69.2
LV mass, g	168.2 ± 58.9
Maximal thickness measured, mm	18.2 ± 5.7
LVEF, %	64.8 ± 11.8
Late enhancement	34 (32.1%)

LVH: left ventricular hypertrophy; AVB: atrioventricular block; HCM: hypertrophic cardiomyopathy; LVEF: left ventricular ejection fraction; LA: left atrial; LV: left ventricular.

Obstruction of the LV outflow tract was present in approximately 40.9% of the patients, resulting in a gradient of 36 ± 36 mmHg. The echocardiographic measures were as follows: interventricular septum thickness, 17 ± 5 mm; posterior wall thickness, 11 ± 3 mm; left atrial diameter, 43 ± 7 mm. Table 1 shows the results of the exercise test and major continuous variables assessed on CMRI.

The screening for mutations for HCM was performed in 83 (79.0%) patients, 28 of whom (26.7%) had one mutation as follows: the *MYBPC3* gene mutation in 20 patients (71.4%); the *TNNT2* gene mutation in 3 (10.7%); the *MYH7* gene mutation in 3 (10.7%); and the *TPM1* gene mutation in 2 (7.1%) patients.

Complex ventricular dysrhythmia episodes were identified in 25 (23.8%) patients on 24-hour Holter.

Regarding primary prevention, according to the 2011 ACCF/AHA recommendations, 38.1% of the patients had indication for ICD implantation (level of evidence class IIa). The device was implanted in 24 (22.9%) patients. It is worth noting that 6 patients refused the device implantation, and 10 patients did not undergo implantation because of their comorbidities.

During the 6-year clinical follow-up, 1 patient received appropriate shock due to ventricular fibrillation (risk score for SCD due to HCM 1.71% - ICD usually not considered). In 25 (23.8%) patients, the ICD recorded ventricular tachycardia (VT) episodes and 3 inappropriate shocks. Ten (9.5%) patients died (6 patients due to heart failure, 1 patient due to ventricular fibrillation, and 3 patients due to neoplasm).

**Figure 1:**
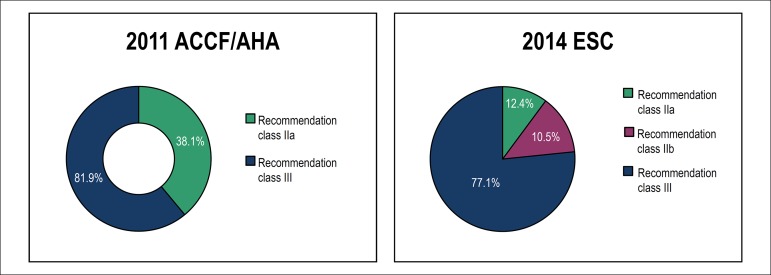
Comparison of risk stratification of SCD due to HCM according to the 2011 versus 2014 recommendations.

According to the 2011 ACCF/AHA recommendations, 38.1% of the patients had indication for ICD implantation (level of evidence class IIa), while 61.9% did not (level of evidence class III) - Figure 1.

According to the 2014 recommendations, the mean risk score for SCD due to HCM in the study population was 3.1±2.7%. Based on that value, the patients were stratified into three risk categories for ICD implantation: 81 (77.1%) patients had a score < 4% (ICD usually not considered - recommendation level III); 11 (10.5%) had a score between 4% and 6% (ICD can be considered - recommendation level IIb); and 13 (12.4%) had a score >6% (ICD should be considered - recommendation level IIa) - Figure 1.

Grouping together the patients classified as 2014 ESC classes IIb and III, 13 (12.4%) patients had recommendation for ICD implantation for primary prevention, while 64 (61.0%) patients did not have that recommendation according to the 2011 and 2014 guidelines. According to the 2011, but not the 2014, guideline, 28 (26.7%) patients had recommendation for ICD implantation. Thus, in 77 (73.3%) patients, the classifications were concordant, but not in 26.7%. The discordant patients were in the same circumstance, that is, according to the 2011 guideline they had indication for ICD implantation for primary prevention, while, according to the 2014 guidelines, ICD implantation would not usually be considered. This is not random, because, of the 28 discordant patients, there were significantly more patients for implantation in 2011 and not in 2014, than vice-versa (p < 0.001 McNemar test).

**Figure 2:**
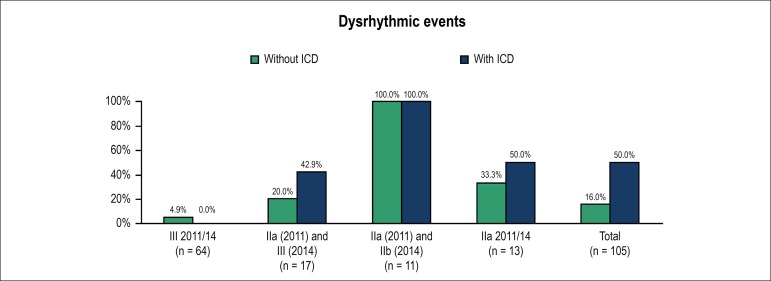
Comparison of the occurrence of dysrhythmic events during clinical follow-up.

After that analysis, four groups of patients were defined, and, by using the exact chi-square test, the occurrence of dysrhythmic events during clinical follow-up was compared between groups - Figure 2.

Regarding the patients classified as recommendation level III according to both guidelines, that is, no indication for ICD implantation, the device was implanted in 3 out of 64 patients. We observed that of the 61 patients who did not undergo ICD implantation, 3 (4.9%) had VT during follow-up. The 3 patients who underwent ICD implantation for primary prevention had no dysrhythmic event. The groups with and without ICD were compared regarding the percentages of events, but no statistical difference was found between them (p = 1.00) - Table 2.

**Table 2:** Comparison of dysrhythmic events in the different groups

Groups				Dysrhythmic events		Total	p
				No	Yes		
III in 2011 and 2014	ICD	No	N %	58 / 95.1	3 / 4.9	61 / 100	1.00
		Yes	N %	3 / 100	0 / 0	3 / 100	1.00
	Total		N %	61 / 95.3	3 / 4.7	64 / 100	1.00
IIa in 2011 and III in 2014	ICD	No	N %	8 / 80.0	2 / 20.0	10 / 100	0.59
		Yes	N %	4 / 57.1	3 / 42.9	7 / 100	
	Total		N %	12 / 70.5	5 / 29.4	17 / 100	0.59
IIa in 2011 and IIb in 2014	ICD	No	N %		7 / 100	7 / 100	1.00
		Yes	N %		4 / 100	4 / 100	
	Total		N %		11 / 100	11 / 100	1.00
IIa in 2011 and 2014	ICD	No	N %	2 / 66.7	1 / 33.3	3 / 100	
		Yes	N %	5 / 50.0	5 / 50.0	10 / 100	
	Total		N %	7 / 53.8	6 / 46.2	13 / 100	1.00
TOTAL	ICD	No	N %	68 / 84.0	13 / 46.2	81 / 100	0.001
		Yes	N %	12 / 50.0	12 / 50.0	24 / 100	
	Total		N %	80 / 76.2	25 / 23.8	105 / 100	0.001

Regarding the group classified as level IIa in 2011 but level III in 2014, of 17 patients, 10 did not undergo ICD implantation, while 7 underwent ICD implantation for primary prevention. Of the 10 who did not undergo ICD implantation, 2 (20.0%) had VT. Of those who had an ICD implanted, 3 (42.9%) had ventricular dysrhythmia during follow-up. The groups with and without ICD were compared regarding the percentages of events, but no significant statistical difference was found between them (p = 0.59) - Table 2.

In the group classified as level IIa in 2011 and IIb in 2014, despite the need for ICD implantation for primary prevention, the device was implanted in 4, but not in 7 patients. In both groups, all patients had dysrhythmic events (p = 1.00). The ICD implantation seems beneficial, but the sample is small - Table 2.

Regarding the patients classified as recommendation level IIa according to both guidelines, that is, indication for ICD implantation for primary prevention, of the total of 13 patients, 3 did not undergo the procedure, while 10 did. Of the 3 patients not undergoing ICD implantation, 1 (33.3%) had VT during follow-up. Of the 10 receiving an ICD, 5 (50,0%) had dysrhythmic events. The groups with and without ICD were compared regarding the percentages of events, but no statistical difference was found between them (p = 1.00) - Table 2.

Of the total population of 105 patients, those who underwent and those who did not undergo ICD implantation for primary prevention were compared regarding the percentages of events. Of the 81 patients who did not receive an ICD, 13 (16.0%) had dysrhythmic events. Of the 24 patients with an ICD, 12 (50.0%) had VT/ventricular fibrillation. Comparing the percentages of events in the two groups, there was a statistically significant difference (p = 0.001) - Table 2.

## Discussion

Our sample of 'real world' patients with HCM had a 22.6% prevalence of ICD implantation. The proportion of patients with HCM and indication for ICD for primary prevention significantly decreased when comparing the 2011 and 2014 guidelines. During clinical follow-up, we detected the presence of complex ventricular dysrhythmia on Holter and/or ICD in some patients, of whom only a minority had a risk score of SCD due to HCM > 6%. In our population, 1 patient with a score < 4%/5 years died due to ventricular fibrillation. According to the literature, in Portugal, no other center has published a study with which we could compare our data and experience.

The gold-standard treatment for primary and secondary prevention of SCD in patients with HCM is ICD implantation, which proved effective in interrupting potentially lethal ventricular tachyarrhythmias, altering the disease's natural history.^1,7^ The efficacy of that therapy has been consolidated since 2000, and has been recently reinforced in a meta-analysis examining the results of 16 studies published between 1998 and 2012, regarding ICD interventions and complications in primary and secondary preventions.^17-22^

The risk stratification of SCD in patients with HCM according to the 2011 ACCF/AHA recommendations was effective in identifying many patients who could benefit from ICD implantation. However, the method proved to be incomplete and some patients without the conventional risk factors were excluded and remained at risk for SCD.^23,24^ Thus, the development of new SCD markers for risk stratification is required.^11^ In 2013, a group of English researchers suggested a new risk score of SCD due to HCM at 5 years. It is a mathematical and statistically complex model.^13^ That score has been rapidly incorporated into the 2014 ESC recommendations as the valid and independent method to select/exclude patients for ICD implantation in primary prevention.^1^

The major objective of any stratification method is its reliability to identify patients at major risk for events, being thus candidates for ICD implantation in primary prevention of SCD. It is worth noting that the new SCD risk model has incorporated arbitrarily two new risk markers (LV outflow tract gradient and left atrial diameter), which had not previously shown to be independent predictors of SCD due to HCM and are not included as risk markers for patients' assessment.^2,10,18^

This study was not aimed at validating (or invalidating) the risk score of SCD due to HCM, but at characterizing the clinical performance of that model individually in a population of Portuguese patients with HCM.

It is worth noting that this analysis showed that the risk model seems to have little sensitivity to identify patients at elevated risk for arrhythmic events and SCD, who, according to the conventional criteria, would be candidates for prophylactic ICD implantation. For example, in the sample of 28 patients with complex dysrhythmic events during the 6-year clinical follow-up, only 4.7% had a risk score > 6%/5 years, which would have justified ICD implantation in primary prevention. In addition, most patients had a score <4%/5 years, that is, no indication for treatment with ICD.

It is worth noting that HCM is a complex pathology, with a spectrum of histological findings and varied and unpredictable clinical manifestations, and a relatively low percentage of SCD.^1,2,10,22,24-29^ Thus, intuitively it would not be expected that the clinical decision individualized for each patient could be based only on a complex mathematical formula, minimizing the fundamental clinical reasoning when facing a patient with HCM.

Being a genetic pathology, some specific mutations might pose a higher risk for SCD. However, it is difficult to determine the existence of a consistent genotype/phenotype correlation, explaining the inability to establish an accurate prognosis based on specific mutations.^4^ Thus, given the inconsistency, they were not included as markers in the current risk model.

However, an important omission in this model is that of quantified late enhancement on CMRI, which several studies have shown to be an independent marker of adverse arrhythmic events (NSVT, VT, ventricular fibrillation) and SCD,^30-34^ even in patients without the conventional risk factors.

Some individuals with HCM can develop LV apical aneurysms, associated with local healing and greater propensity to potentially lethal arrhythmias and SCD,^35^ in addition to heart failure with systolic dysfunction^36^ and coronary atherosclerotic disease,^37^ which are not contemplated in the risk score of SCD. Some prediction inconsistency of the new risk model might be related to the inclusion of some variables, such as syncope, NSVT, left atrial diameter and LV outflow tract obstruction gradient (non-static variables).^11,24,38,39^

The strategy of conventional risk stratification prioritizes SCD prevention in patients with HCM *versus* excessive ICD implantation. On the contrary, the new risk score seems to identify many patients at low risk, who are not candidates for ICD implantation. There is, thus, a significant reduction in the number of devices implanted, but it seems at the cost of misclassifying some patients at high risk for arrhythmic events and SCD.

### Study limitations

Our study has some limitations, because it is based on a single center, with a reduced number of patients and events. However, calculating the sample power ensured that the number of patients was sufficient to draw conclusions. As in any retrospective study, we were limited by the information available in the patients' medical records.

## Conclusion

Hypertrophic cardiomyopathy is a complex pathology, with a wide and unpredictable clinical spectrum.

According to our data, the current risk stratification model seems to reduce the proportion of patients with indication for ICD implantation. It is worth noting that the decision based on a mathematical model that minimizes the individual clinical reasoning seems a little reliable strategy to identify patients at risk for events due to HCM.
